# The Alexandria library, a quantum-chemical database of molecular properties for force field development

**DOI:** 10.1038/sdata.2018.62

**Published:** 2018-04-10

**Authors:** Mohammad M. Ghahremanpour, Paul J. van Maaren, David van der Spoel

**Affiliations:** 1 Uppsala Centre for Computational Chemistry, Science for Life Laboratory, Department of Cell and Molecular Biology, Uppsala University, Husargatan 3, Box 596, SE-75124 Uppsala, Sweden

**Keywords:** Physical chemistry, Cheminformatics, Databases

## Abstract

Data quality as well as library size are crucial issues for force field development. In order to predict molecular properties in a large chemical space, the foundation to build force fields on needs to encompass a large variety of chemical compounds. The tabulated molecular physicochemical properties also need to be accurate. Due to the limited transparency in data used for development of existing force fields it is hard to establish data quality and reusability is low. This paper presents the Alexandria library as an open and freely accessible database of optimized molecular geometries, frequencies, electrostatic moments up to the hexadecupole, electrostatic potential, polarizabilities, and thermochemistry, obtained from quantum chemistry calculations for 2704 compounds. Values are tabulated and where available compared to experimental data. This library can assist systematic development and training of empirical force fields for a broad range of molecules.

## Background & Summary

Chemical space is spanned by all possible molecules that are energetically stable^1^. The to date largest generated database (GDB-17) contains 166.4 billion molecules of up to 17 atoms of H, C, N, O, S, and halogens^2^ (a more workable representative subset containing 10 million compounds was published recently as well^3^). Computational chemistry has enabled us to virtually explore and exploit the chemical space by predicting physicochemical properties of its compounds^4^. This has helped chemical biologists, for example, to identify bioactive regions of the chemical space^4–6^. The main challenge when dealing with large numbers of compounds is to predict properties with good accuracy at moderate computational cost.

Compounds in the chemical space may vary in size, they may be organic or inorganic, including synthetic- and bio-polymers^7,8^. In addition, the chemistry of life^9^ happens in the liquid phase, which implies that we need to explore the properties of a large range of compounds in at least the gas- and the liquid phases. Hence, the practical tool for navigating chemical space is atomistic molecular simulations based on empirical force fields.

Many areas of materials science and drug discovery have benefited from the application of empirical force fields. High-throughput virtual screening is a promising approach, that has led to the discovery of new materials and drug-like compounds^10^. However, making accurate prediction of properties of molecules from different parts of the chemical space is yet to be achieved since force fields are not readily transferable from one chemical category to another. In other words, the reliability and the applicability of force fields in practice depends on the chemical composition of the compounds under investigation. The main reason is that empirical force fields are in essence derived using supervised machine learning algorithms that can learn from and make predictions on the available data. The quality of the data and the diversity of the molecules in the database determine the domain of accuracy and reliability of the resulting force fields. Therefore, data quality should be carefully considered when developing force fields. However, the databases used for optimizing force fields are rarely published and when they are made available they are in a format that is difficult to use in data-mining. As a result, it is difficult to assess the underlying data quality for the existing force fields.

Several resources are available providing experimental data for physicochemical properties. For instance, the National Institute of Standard and Technology^11,12^ and the Design Institute for Physical Properties^13^ have collected large amounts of experimental molecular properties measured during many decades of research. Due to the size of chemical space there is experimental data only for a small fraction of molecules-most of these databases contain less than ten thousand compounds. In addition, most of the data provided for molecular properties is old and the original sources may not be readily accessible. It would be prohibitively expensive to experimentally determine all the properties of interest for even a small fraction of designed compounds from, e.g., GDB-17. For this reason, the dissemination of quantum chemistry data for a set of assorted molecules is very useful to accelerate progress in empirical force fields. For example, Ramakrishnan *et al.*^14^ have provided a quantum-chemistry database of molecular geometries and properties for 134,000 molecules at the B3LYP/6-31G(2df,p) level of theory, for development of machine learning tools. Moreover, the ANI-1 database provides off-equilibrium density functional theory (DFT) calculations for 57,454 organic molecules up to 8 heavy atoms including H, C, N, and O^15^. Other databases are available as well at both high^16,17^ and low levels of theory^18^. These resources containing quantum-chemical molecular properties are of interest for optimization of molecular mechanics potentials for small compounds by facilitating the development of machine learning strategies for predicting molecular properties^19,20^.

This paper presents the Alexandria library, an open and freely accessible database of quantum-chemically optimized molecular structures and properties of 2704 compounds for empirical force field development. The name “Alexandria” was adopted to highlight that we aim to collect “all” knowledge in the world, old and new, on molecular properties, just like the legendary library of Alexandria, since it has been established that availability of data rapidly declines with time^21^. The library could also be used for evaluation of density functionals and development of semi-empirical quantum methods. The compounds belong to more than thirty different chemical categories containing functional groups that are common in biomolecules and drug-like compounds. They are predominantly made up of C, H, N, O, Si, P, S, and halogens covering the elements of the GDB-17 chemical space. The library also provides data for some inorganic compounds and metals. The molecular properties provided here are enthalpy of formation, heat capacity, absolute entropy, zero-point vibrational energy, vibrational frequencies, electric moments up to hexadecapole, and polarizability, all in the gas phase. Thermochemistry calculations are in part based on our previous work^22^. In addition, the electrostatic potential on a grid around the compound and the partial atomic charges (Mulliken charges^23^, Hirshfeld charges^24^, ESP charges^25^, and CM5^26^) are computed for each molecule. Where data is available we compare the quantum chemical calculations to experimental data. For transparency, we make the input files used to perform the quantum chemical calculations as well as all the output files available. This allows for testing the reproducibility of the quantum chemical data provided in the Alexandria library.

## Methods

Initial structures were downloaded from the PubChem^27^ and the ChemSpider^28^ databases for most of the molecules. The downloaded structures were checked for missing hydrogens and the presence of 3D coordinates. The rest of molecules were generated by Avogadro^29^ or Molden^30^ softwares and their structures were minimized before performing quantum calculations. Quantum chemistry calculations were performed using the Gaussian 09^31^ and Gaussian 16 (ref. 32) set of programs. The B3LYP level of density functional theory^33–36^ was used in combination with the aug-cc-pVTZ basis set^37–39^ to optimize molecular geometries and to calculate frequencies, electric moments, polarizabilities, electrostatic potential surface and the corresponding partial atomic charges for each molecule (Table 1). The Merz-Kollman scheme, as implemented in Gaussian 16^32^, was used to generate the grids around the molecule in order to calculate the electrostatic potential surface^40,41^. The B3LYP functional was combined with the aug-cc-pVTZ-PP basis set^42^ to take relativistic pseudopotentials into account for compounds containing iodine. For reference, the same calculations were also performed at the HF/6-311G** (refs 43–46) level of theory (Table 1), which is similar to widely used methods to calculate partial atomic charges for virtual screening of large chemical libraries. The G2, G3, G4 (refs 47–51), CBS-QB3 (refs 52,53), W1U, and W1BD (ref. 54) methods were used to calculate enthalpy of formation (Δ_*f*_*H*^0^), heat capacity at constant volume (*C*_*V*_), and absolute entropy (*S*^0^) at room temperature (Table 1). The Weizmann family of methods was used on a subset of about 600 compounds only, due to computational cost. The procedure of thermochemistry calculations has been explained in detail in our previous work^22^.

The OpenBabel program (version 2.4.1)^55^ was used to determine the number of rotatable bonds based on the optimized geometry for each molecule. The results, wherever possible, were compared to the PubChem database^27^ to check for consistency and manually curated in case of discrepancies. We here count bonds as rotatable if they increase the number of unique conformations. In our previous work^22^ we introduced an OpenBabel tool obthermo to extract thermochemistry data from Gaussian^31^ output files (with the aid of library of atomization energies, provided in OpenBabel (version 2.4.1). This open source tool contributes to our aim to make the data provided here accessible to other workers in the field.

## Data Records

The Alexandria library contains the input (.com) and the output (.log) files in GNU-zip compressed format (.gz) of quantum chemical calculations performed using Gaussian 09 (ref. 31) or Gaussian 16 (ref. 32) (Data Citation 1). All compounds are provided in a single Chemical Markup Language (CML) and in a single Tripos Mol2 (.mol2) file as well. The .mol2 file contains the optimized geometries at the B3LYP/aug-cc-pVTZ level of theory, the atomic partial charges computed by the ESP fitting algorithm, and the bond information. The molecular electrostatic potential surface used to fit the atomic partial charges is also provided in (compressed) XML files for each compound. This must be used in conjunction with the corresponding coordinates of the compound, that can be extracted from the Gaussian log files using OpenBabel. SMILES fingerprints were also generated for all molecules using the OpenBabel software (version 2.4.1)^55^ and stored in a .smiles file.

For each quantum chemical method, a table is provided in a .csv file (comma-separated value, however since both compound names and InChI identifiers contain comma's, we use the pipe symbol '|' as a separator). The files include the compound information (Table 2), the calculated and the experimental values of the molecular dipole moment, polarizability and thermochemistry results. These tables can be read using either commercial or open source spreadsheet software but they can also be processed by scripting languages. Further molecular properties are available in the Gaussian log files that can be extracted by OpenBabel software (version 2.4.1)^55^ or other software.

## Technical Validation

### Experimental Data

The experimental results used for the validation of quantum chemistry calculations are taken from several sources^13,56–60^. In some cases the values were cross referenced against the original publication to check for transcription errors. For compounds where multiple values for the same property were found, the average and the standard deviation of the values were taken to be the reference value and the uncertainty, respectively^22^. It should be noted that we found approximately 240 suspected errors in the experimental data in our previous work^22^ which are excluded from comparisons in this study. It can obviously not be excluded that there are more errors in the experimental reference data leading to less good agreement with calculations.

### Quantum Chemical Calculations

We have previously benchmarked and validated a number of standard quantum thermochemistry methods used to build the Alexandria library and shown that the G4 theory is a good compromise for thermochemistry calculations in comparison to the other methods^22^. Therefore, we here focus on the validation of optimized geometries, molecular polarizability, and dipole moments.

The optimized geometries were validated by comparing the StdInChI generated from each optimized geometry to the StdInChI obtained from PubChem database^27^. Moreover, the StdInChI obtained from the initial structure is compared to the StdInChI generated from the optimized structure confirming that both the initial and the optimized geometries correspond to the same compound^14,61^. 40 compounds out of 2704 did not pass this test, because StdInChI representations are not unique and thus the generation of StdInChI from Cartesian coordinates is error prone. This problem has been discussed in detail elsewhere^14^. Here, these 40 compounds were validated manually.

**Figure 1:**
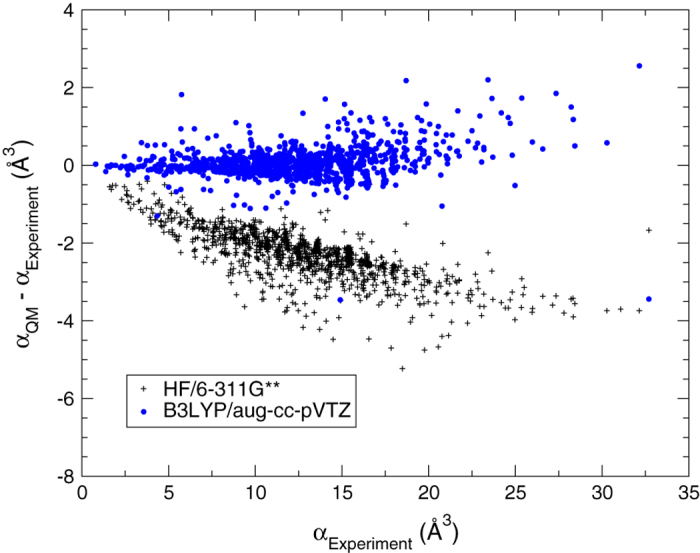
Residual plot for the isotropic polarizability *α* as calculated at two levels of theory.

**Figure 2:**
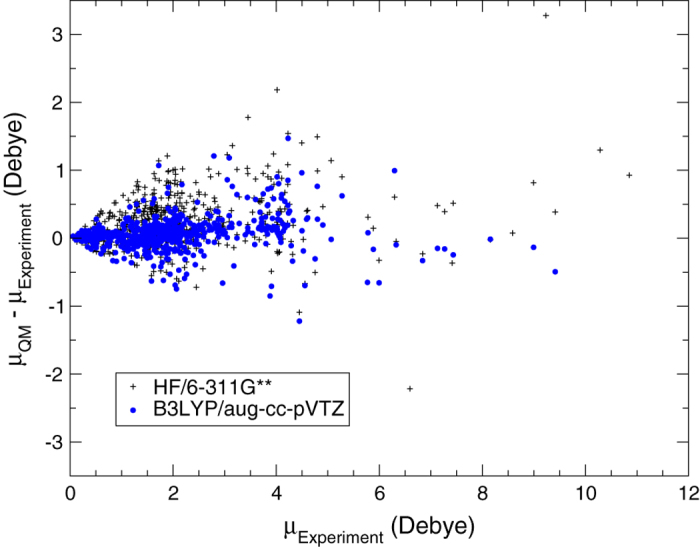
Residual plot for the dipole moment *μ* as calculated at two levels of theory.

DFT calculations of molecular dipole moment and isotropic polarizability were validated by comparing to experiments. The B3LYP/aug-cc-pVTZ level of theory^34,37–39,62^ showed much lower RMSD than the HF/6-311G^**^ level of theory^63,64^ for isotropic polarizability (Table 3). Hartree-Fock calculations with the 6-311G^**^ basis set systematically underestimate the molecular isotropic polarizability (Fig. 1). However, the distribution of the residuals is homogenous for the B3LYP calculations with the aug-cc-pVTZ basis set (Fig. 1), indicating that B3LYP/aug-cc-pVTZ yields reliable predictions of the isotropic polarizability. The comparison between experimental and quantum-mechanical dipole moments was done for rigid molecules only, because the experimental dipole moment of flexible molecules, which represents an average over the accessible conformations at the experimental temperature, is not comparable to the computed dipole moment of a single conformation at zero Kelvin. Therefore flexible molecules were excluded from the statistics of the calculated dipole moments listed in Table 3 and from the residual plot presented in Fig. 2. In this work, a molecule is considered flexible if it has at least one rotatable bond. The RMSD from experimental dipole moments is found to be ≈ 0.2D higher for HF/6-311G^**^ than for B3LYP/aug-cc-pVTZ (Table 3). Fig. 2 also shows that B3LYP with the aug-cc-pVTZ basis set is accurate enough to reproduce experimental dipole moments, and hence, to predict values for molecules where there is no experimental data, at least for those compound categories in this data set.

The experimental and quantum chemical data provided in this paper also allow performing systematic analyses of molecular properties. Such analyses aid in understanding the relation between the chemical composition and the physicochemical properties of molecules. The variation of the experimental isotropic polarizability between different chemical formulae is small (Table 4). The mean signed errors (MSE) show that the B3LYP/aug-cc-pVTZ level of theory slightly underestimates the isotropic polarizability for most of the chemical formulas listed in Table 4. The standard deviation obtained from the experimental thermochemistry data show that the standard entropy (Table 5) and the heat capacity at constant volume (Table 6) can be predicted quite accurately by the chemical formula, while this does not hold for the enthalpy of formation (Table 7). The MSE values show that the G4 theory underestimates the entropy and heat capacity at constant volume (Tables 5 and 6), however, it overestimates the enthalpy of formation for most of the chemical formulas (Table 7).

## Usage Notes

Programs like Molden^30^, Avogadro^29^ and GaussView can be used to visualize and analyze quantum chemical calculations. Moreover, the obthermo^22^ program implemented in the OpenBabel program package (version 2.4.1)^55^ extracts enthalpy of formation, heat capacity at constant volume, and absolute entropy from the Gaussian 09 (ref. 31) and Gaussian 16 (ref. 32) log files. It can also be used to estimate the heat capacity at constant pressure from the calculated heat capacity at constant volume and the temperature derivative of the second virial coefficient, which must then be specified by the user as the input to the program^22^.

## Additional information

**How to cite this article**: Ghahremanpour, M. M. *et al.* The Alexandria library, a quantum-chemical database of molecular properties for force field development. *Sci. Data* 5:180062 doi: 10.1038/sdata.2018.62 (2018).

**Publisher’s note**: Springer Nature remains neutral with regard to jurisdictional claims in published maps and institutional affiliations.

## Supplementary Material



## Figures and Tables

**Table 1:** The number of calculations for each quantum-chemical method in the library.

**Method**	***N***_***qm***_
B3LYP/aug-cc-pVTZ	2500
CBS-QB3	2179
G2	2096
G3	2090
G4	2091
HF/6-311G**	2537
W1BD	705
W1U	606
G2, G3, G4, CBS-QB3, W1BD, and W1U were used to calculate thermochemical properties. The HF/6-311G** and B3LYP/aug-cc-pVTZ levels of theories were used to optimize the molecular geometries, determine the electric moments and polarizability, molecular electrostatic potential map, atomic partial charge, vibrational frequencies, and the zero-point vibrational energy. Note that not all calculations have been done for all compounds, therefore some numbers above are lower than the total number of compounds.	

**Table 2:** Compound information provided in the repository.

	**Information**
1	IUPAC name
2	Formula
3	Total charge
4	Multplicity
5	CAS number
6	ChemSpider ID (CSID)
7	PubChem ID (CID)
8	Number of rotatable bonds
9	StdInChI
10	InChIKey

**Table 3:** Root mean square deviation (RMSD) from experiment for polarizability *α* and dipole moment *μ* for compounds where calculations were done at both levels of theory.

**Property**	**N**	HF/6-311G**	**B3LYP/aug-cc-pVTZ**
*α* (Å^3^)	1198	2.39(0.004)	0.43(0.006)
*μ* (D)	542	0.48(0.002)	0.30(0.003)
The RMSD and its error bar are obtained by bootstrapping with 100 iterations. N is the number of compounds, which is limited by the availability of experimental data.			

**Table 4:** Chemical space analysis of polarizability *α*.

**Formula**	**N**_***exp***_	***α*** **(Å**^**3**^**)**	**N**_***qm***_	**RMSD (Å**^**3**^**)**	**MSE**
C_4_H_6_O_2_	8	9(0.3)	6	0.1	0.1
C_4_H_8_O_2_	9	9(0.2)	9	0.2	0.0
C_4_H_10_O_2_	9	9(0.1)	8	0.1	0.0
C_5_H_8_	12	10(0.5)	10	0.2	0.2
C_5_H_10_	10	10(0.3)	9	0.2	−0.2
C_5_H_10_O_2_	11	11(0.1)	11	0.1	−0.1
C_5_H_10_O	10	10(0.1)	9	0.1	−0.1
C_5_H_12_O	11	11(0.2)	10	0.3	−0.2
C_6_H_10_	35	12(0.5)	28	0.3	0.1
C_6_H_12_O_2_	10	13(0.0)	8	0.1	−0.0
C_6_H_12_	30	12(0.3)	29	0.2	−0.2
C_6_H_12_O	11	12(0.3)	9	0.2	−0.1
C_6_H_14_O	14	12(0.1)	13	0.2	−0.1
C_7_H_9_N	12	14(0.3)	7	0.1	0.1
C_7_H_12_	23	13(0.2)	21	0.2	−0.1
C_7_H_14_	44	13(0.3)	41	0.2	−0.2
C_7_H_14_O	17	14(0.2)	8	0.2	−0.2
C_7_H_16_	9	14(0.1)	9	0.3	−0.3
C_8_H_10_O	9	15(0.1)	5	0.2	0.1
C_8_H_11_N	9	16(0.4)	5	0.4	0.2
C_8_H_16_	113	15(0.3)	109	0.3	−0.2
C_8_H_18_	18	15(0.1)	16	0.4	−0.4
C_9_H_10_	8	16(0.4)	6	0.7	0.6
C_9_H_12_	8	16(0.1)	7	0.1	0.1
C_9_H_18_	31	17(0.2)	25	0.4	−0.4
C_9_H_18_O	9	17(0.1)	2	0.5	−0.5
C_9_H_20_	16	17(0.1)	6	0.2	−0.2
C_10_H_14_	19	18(0.1)	12	0.2	0.1
C_10_H_22_	14	19(0.1)	3	0.1	−0.0
Number of compounds with experimental data N_*exp*_, experimental average *α* for all isomers with standard deviation within brackets, number of compounds with B3LYP/aug-cc-pVTZ calculations N_*qm*_, root mean square deviation (RMSD) between calculation and experiment, mean signed error (MSE) in calculations.					

**Table 5:** Chemical space analysis of standard entropy S^0^.

**Formula**	**N**_***exp***_	**S**^**0**^ **(J/mol K)**	**N**_***qm***_	**RMSD (J/mol K)**	**MSE**
C_4_H_8_O_2_	12	349(29.0)	12	15.9	8.1
C_4_H_10_O_2_	8	384(11.8)	7	16.5	−8.9
C_5_H_8_	11	318(16.0)	10	4.8	1.1
C_5_H_10_	10	327(18.1)	10	8.1	0.1
C_5_H_10_O_2_	11	394(11.7)	9	10.4	4.1
C_5_H_12_O	12	381(11.5)	11	7.0	−3.4
C_6_H_10_	20	354(17.7)	17	8.1	0.6
C_6_H_12_O_2_	10	444(21.2)	10	18.6	−1.8
C_6_H_12_	19	368(20.6)	19	5.8	−0.9
C_6_H_12_O	8	402(30.2)	5	8.8	−0.9
C_6_H_14_O_2_	8	461(22.2)	4	17.1	−9.7
C_6_H_14_O	14	424(12.2)	9	8.1	−2.6
C_7_H_9_N	9	355(6.8)	7	10.2	3.4
C_7_H_12_	23	375(26.3)	23	9.5	−2.5
C_7_H_14_	20	395(29.9)	19	8.1	−2.7
C_7_H_14_O	15	417(33.4)	6	17.0	−0.1
C_7_H_16_	9	408(14.9)	9	9.4	5.3
C_8_H_10_O	12	395(5.2)	10	10.2	−8.0
C_8_H_16_	31	414(37.2)	30	7.9	−0.6
C_8_H_18_	18	441(18.9)	17	6.2	1.9
C_9_H_10_	8	382(16.6)	7	9.8	−1.2
C_9_H_12_	10	392(9.2)	7	7.3	3.7
C_9_H_18_	9	463(41.8)	2	2.5	−2.5
C_9_H_20_	16	470(25.7)	6	17.3	12.9
C_10_H_14_	20	428(10.2)	5	9.3	6.0
C_10_H_22_	14	522(23.3)	2	8.1	7.6
Number of compounds with experimental data N_*exp*_, experimental average S^0^ for all isomers with standard deviation within brackets, number of compounds with G4 calculations N_*qm*_, root mean square deviation (RMSD) between calculation and experiment, mean signed error (MSE) in calculations.					

**Table 6:** Chemical space analysis of heat capacity at constant volume C_v_.

**Formula**	**N**_***exp***_	**C**_**v**_ **(J/mol K)**	**N**_***qm***_	**RMSD (J/mol K)**	**MSE**
C_4_H_8_O_2_	9	97(8.7)	9	5.2	0.8
C_4_H_8_O	8	88(7.9)	7	4.9	−3.6
C_5_H_8_	12	91(7.4)	11	3.9	−2.8
C_5_H_10_	10	97(8.6)	10	5.7	−2.9
C_5_H_10_O_2_	11	126(2.9)	9	9.3	−6.9
C_5_H_12_O	12	127(4.3)	11	7.6	−6.4
C_6_H_10_	16	110(11.1)	13	5.5	−2.0
C_6_H_12_O_2_	9	148(2.4)	9	5.6	−3.3
C_6_H_12_	19	121(9.2)	19	8.0	−4.5
C_6_H_14_O	11	149(2.0)	7	10.0	−9.0
C_7_H_9_N	9	117(2.7)	7	3.6	−0.4
C_7_H_12_	23	131(11.4)	23	6.5	−4.4
C_7_H_14_	19	137(9.8)	18	6.2	−5.2
C_7_H_14_O	14	151(10.8)	6	11.6	−5.5
C_7_H_16_	8	156(2.7)	8	8.6	−7.6
C_8_H_10_O	12	143(8.6)	10	8.0	−5.4
C_8_H_16_	18	156(10.7)	18	8.0	−7.1
C_8_H_18_	18	179(2.8)	17	10.4	−9.7
C_9_H_10_	8	133(4.8)	7	3.2	−2.2
C_9_H_12_	10	143(4.3)	7	3.8	−1.3
C_9_H_20_	16	201(3.4)	6	14.7	−14.6
C_10_H_14_	20	170(3.8)	5	5.3	−4.4
C_10_H_22_	14	223(2.5)	2	17.6	−17.5
Number of compounds with experimental data N_*exp*_, experimental average C_v_ for all isomers with standard deviation within brackets, number of compounds with G4 calculations N_*qm*_, root mean square deviation (RMSD) between calculation and experiment, mean signed error (MSE) in calculations.					

**Table 7:** Chemical space analysis of enthalpy of formation Δ_*f*_H^0^.

**Formula**	**N**_***exp***_	**Δ**_***f***_**H**^**0**^ **(kJ/mol)**	**N**_***qm***_	**RMSD (kJ/mol)**	**MSE**
C_4_H_8_O_2_	12	−379(63.9)	12	14.0	9.3
C_4_H_8_O	8	−172(42.3)	7	3.5	−0.8
C_4_H_10_O_2_	10	−404(66.4)	9	14.6	10.9
C_5_H_8_	12	114(39.3)	11	3.2	1.7
C_5_H_10_	12	−23(21.7)	12	5.6	2.0
C_5_H_10_O_2_	11	−457(36.2)	9	10.5	2.6
C_5_H_10_O	9	−244(13.6)	8	8.4	3.8
C_5_H_12_O	12	−293(21.3)	11	7.8	−0.7
C_6_H_10_	20	68(41.8)	17	7.5	2.9
C_6_H_12_O_2_	11	−470(56.7)	11	19.1	10.5
C_6_H_12_	28	−52(23.7)	28	8.5	3.3
C_6_H_12_O	8	−267(33.9)	5	2.7	−1.1
C_6_H_14_O_2_	9	−466(34.4)	4	17.8	11.9
C_6_H_14_O	14	−317(19.3)	9	6.5	0.7
C_7_H_9_N	9	69(15.2)	7	7.1	1.3
C_7_H_12_	28	31(54.9)	26	8.5	−0.9
C_7_H_14_	44	−88(23.0)	43	7.2	2.6
C_7_H_14_O	15	−316(30.4)	6	23.7	14.6
C_7_H_16_	9	−197(6.3)	9	3.2	0.7
C_8_H_10_O	12	−148(17.9)	10	10.6	3.4
C_8_H_16_	104	−111(25.3)	102	6.3	1.8
C_8_H_18_	18	−217(5.0)	17	7.7	4.9
C_9_H_10_	8	113(21.0)	7	3.0	−2.3
C_9_H_12_	10	27(63.1)	7	3.4	−3.3
C_9_H_18_	9	−146(41.0)	2	22.1	18.8
C_9_H_20_	16	−237(6.5)	6	25.8	19.9
C_10_H_14_	20	−27(8.2)	5	15.0	6.1
C_10_H_22_	14	−260(7.8)	2	3.4	−3.4
Number of compounds with experimental data N_*exp*_, experimental average Δ_*f*_H^0^ for all isomers with standard deviation within brackets, number of compounds with G4 calculations N_*qm*_, root mean square deviation (RMSD) between calculation and experiment, mean signed error (MSE) in calculations.					
